# Examination of the Pupils.—IV

**Published:** 1909-05-01

**Authors:** 


### Medicine.

### EXAMINATION OF THE PUPILS.—IV.

### THE LIGHT REFLEX (*continued*).

It is clear that there may be a blockage in the reflex arc in any of the following situations:—

(1) The retina; (2) the optic disc; (3) the optic nerve; (4) the optic chiasma; (5) the optic tract; (6) the nuclear cells of the third nerve, pupillary portion; (7) the third nerve, pupillary fibres; (8) the iris.

In order to facilitate the discussion, it will be assumed in each case that the right side is affected, and that the left side is healthy; and also, although it is quite common for the lesions to be partial, they will be assumed to be complete.

(1) The retina; (2) the optic disc; (3) the optic nerve. These may be taken together because the effects of total lesions of each of them upon the pupillary light reflex are identical. Light thrown into the right eye will cause no contraction of either pupil, whilst light thrown into the left eye will cause contraction not only of the left pupil, but also of the right. There will, of course, be complete blindness of the right eye.

(4) The optic chiasma: Here the lesion cannot, as a rule, be called either right or left, because it is nearly always median. A tumour of the pituitary body is a well-known cause of it. If large enough, the tumour may destroy the whole chiasma, causing total blindness of both eyes and total loss of light reflex in both eyes. In an earlier stage, however, the decussating fibres alone become affected. Light thrown into the right eye will cause contraction of the right pupil, but not of the left—or very much less of the left; and light thrown into the left eye will cause contraction of the left pupil, but little or none of the right.

The above is only true, however, when the light is sufficiently abundant to fall upon all parts of the retina. If a single small pencil of rays is used, owing to blindness of the nasal halves of the retinæ (bilateral temporal hemianopia) there would be no contraction even of the pupil of the same side if the rays fell only on the nasal half of the retina that was being tested. This, however, is a refinement that will be obvious to all who think the matter out.

(5) The right optic tract contains fibres coming from the temporal half of the right retina and from the nasal half of the left retina. A complete lesion of it will therefore cause blindness of these, and consequently the patient will be unable to see anything to the left of him when his eyes are directed straight forward. As regards the pupil reflex, ordinary daylight entering the right eye will cause little contraction of the right pupil, but quite a good one of the left; whereas light entering the left eye will cause a good contraction of the left pupil and little or none of the right.

If pencils of light are used in carrying out the test, and the pencil is allowed to fall upon the temporal half of the right retina, or upon the nasal half of the left retina, there will be no contraction of either pupil; if it falls upon the nasal half of the right retina, the left pupil may contract more briskly than the right; and if it falls upon the temporal half of the left retina, the left pupil may contract well, and the right not at all.

(6) When there is a generalised degeneration of the nuclear cells of the third nerve, there will be a corresponding paralysis both of the external and of the internal muscles of the eyeball—external and internal ophthalmoplegia. One sees this condition sometimes as the result of chronic alcoholism and syphilis. Better known than this, however, is the condition of Argyll-Robertson pupil; whilst the external movements of the eyeball may remain perfect, there is complete loss of the pupillary light reflex, but at the same time the pupils still contract quite well when the eyes are converged as in the act of accommodation. It is believed that this peculiar anomaly is due to degeneration of only those nuclear cells of the third nerve which are concerned in the movements of the pupil, all the other adjacent cells in the nucleus remaining intact. The condition is best seen in locomotor ataxy—so much so that there must always be some hesitation in diagnosing a case as one of tabes dorsalis unless there is what is generally spoken of as "an Argyll-Robertson pupil." It is important to remember, however, that about one case in ten of locomotor ataxy has normal pupillary reactions; and conversely there are certain persons who have had syphilis and who have an Argyll-Robertson pupil without ever developing symptoms of locomotor ataxy.

(7) If the pupillary fibres of the third nerve could be completely destroyed without injuring the other fibres in the nerve, the result as regards the pupillary reactions to light would be precisely similar to that of the focal nuclear cell degeneration upon which the Argyll-Robertson pupil depends. A focal lesion in the nerve is infinitely rarer than a focal lesion in its nucleus, however. A loss of light reflex due to a lesion in the third nerve itself is therefore nearly always associated with other signs of third nerve paralysis, such as ptosis, or strabismus, from ophthalmoplegia externa. It is possible, however, to have no ophthalmoplegia externa and yet to have complete ophthalmoplegia interna. Post-diphtheritic neuritis sometimes picks out just those fibres whose absence leads to ophthalmoplegia interna. Another cause of the condition is subacute alcoholism.

In testing the contractility of the pupil on convergence, the patient is asked to look steadily at some fixed object as far away from him as possible; he is then suddenly asked to look at the observer's finger or some other object such as a match, held about five inches in front of the nose. In accommodating to look at the near object a powerful effort of convergence is made, and under normal conditions the pupil contracts briskly and considerably in association with this voluntary act of convergence.

Presence of this power to contract upon accommodation when contractility to light is lost constitutes the Argyll-Robertson pupil discussed above. Complete ophthalmoplegia interna includes loss of contractility both to light and to accommodation, as also already described. It only remains to mention first, that it has been stated that in some cases of diabetes mellitus the pupil may be unable to react to accommodation though it still reacts to light—a statement that is not borne out by the facts in the great majority of diabetic patients; secondly, there is sometimes an inability to converge the eyeballs in the subjects of exophthalmic goitre, in which case the pupil may or may not contract when the effort is being made; and thirdly, there is a peculiar reaction of the pupil in some alcoholic subjects. The pupil may seem at first to contract properly both to light and to accommodation; but if a careful observation is made, it will be seen to maintain its first contraction for but a very short time only, thereafter dilating again even to a greater diameter than before, and sometimes contracting and dilating through a short series of alternations which might well merit the title of the "oscillatory pupil reaction."

